# *In-vitro* analysis of the microbicidal activity of 6 contact lens care solutions

**DOI:** 10.1186/1471-2334-12-241

**Published:** 2012-10-03

**Authors:** Claudia Hildebrandt, Daniela Wagner, Thomas Kohlmann, Axel Kramer

**Affiliations:** 1 Institute of Hygiene and Environmental Medicine, University Medicine Greifswald, Greifswald, Germany; 2 Institute of Community Medicine, University Medicine Greifswald, Greifswald, Germany

**Keywords:** Contact lens care solutions, Microbicidal activity, EN ISO 14729

## Abstract

**Background:**

Contact lens-related infections are often associated with inadequate contact lens hygiene, and therefore, contact lens care products should be able to sufficiently minimise the amount of pathogens that are responsible for these infections. In 2001, the EN ISO 14729 was introduced to ensure adequate disinfection efficacy of contact lens care solutions, but this norm has recently been criticised.

**Methods:**

In this study, six frequently used contact lens care solutions were retested according to the Stand Alone Test of the EN ISO 14729 (2001). The Stand Alone Test is a quantitative suspension test. In addition, the products were tested in a modified setting adding an organic load. The load was a mixture of human blood serum, lysozyme, and mucine, which resembles tear fluid.

**Results:**

The criteria of the Stand Alone Test recommended in EN ISO 14729 were only met by Aosept Plus. This 3% hydrogen-peroxide-based contact lens care solution attained a reduction factor of > 5 log units for bacteria and > 4 for fungi in all cases. Two further contact lens care solutions, Blue Vision and Optifree Replenish, met the criteria of a reduction factor of > 3 log units for bacteria and > 1 log unit for fungi, but only in the presence of artificial tear fluid. The three remaining products did not exhibit adequate disinfecting efficacy, at least against one of the tested microorganisms.

**Conclusions:**

Through the observation that the artificial tear fluid used in this study influences the disinfecting efficacy of contact lens care solutions, especially that of multi-purpose solutions, in a different way than does albumin, mucine, or even the organic load suggested in EN ISO 14729, it becomes obvious that the test conditions in the EN ISO 14729 should be revised in order to create more realistic conditions, e.g., by using a more realistic artificial tear fluid. Furthermore, we suggest adapting the EN ISO 14729 to the European test hierarchy for chemical disinfectants and antiseptics, which consists of three test phases and also requests meeting stricter criteria in order to pass the test. Unless the test conditions guarantee a sufficient reduction of potential pathogens, the risk of contact lens-related microbial keratitis and other infections will remain for the users.

## Background

In developed countries, contact lens (CL) wear, especially hydrogel CL wear, is the most common risk factor for microbial keratitis [1-10] and has steadily increased over the past decades [11]. In the aetiology of CL-associated microbial keratitis, Pseudomonas spp. [11,12] dominate, followed by other bacteria species, fungi, and Acanthamoeba [5,11-29]. In many cases, infections are initiated by the CL wearers themselves. One of the major risk factors is the inadequate hygiene in handling CLs and their accessories [1,30-32]. Numerous studies have concluded that up to 90% of CL wearers are non-compliant with at least some of their CL care regimen [33-36]. This non-compliance is thought to result, for example, from the lack of understanding proper CL care procedures by the CL wearers [33], or from misinformation and misinterpretation of practitioners’ instructions [36].

In the light of these findings, it is essential that CL care products, especially CL care solutions, should be designed to sufficiently decrease the amount of potential pathogens in order to minimise the risk of CL-related infections. CL care products should ideally have a certain level of ‘excess efficacy’, or safety margin [36,37]. Unfortunately, different CL care solutions do not even provide a minimum of safety for the wearer and had to be taken off the market as they were associated with CL-related microbial keratitis [11,38-44]. In addition to these issues, numerous studies have shown that CLs, CL cases and CL care solutions can be loaded with up to 10^7^ colony forming units (CFU) of bacterial, amoebic and even viral pathogens [18,25,45,46].

To improve the situation, the EN ISO 14729 [47] was introduced in 2001 to ensure the disinfection efficacy of CL care solutions. CL care solutions are required to be tested according to this harmonised norm, as they are classified as medical devices Class IIb according to 93/42/EEC [48] rule 15: All devices intended specifically to be used for disinfection, cleaning, rinsing or, when appropriate, hydrating contact lenses are in Class IIb.

However, the EN ISO 14729 was designed with the assumption that the CL wearers perform an adequate rub-and-rinse regimen [49]. Nevertheless, especially for CL wearers who do not comply with their CL care regimen, it is crucial that CL care solutions not only meet the EN ISO Stand Alone primary criteria, but also exceed them under more realistic conditions [50-52]. Therefore, various studies have been conducted on the basis of EN ISO 14729 with an added organic load, with the result that most products failed, especially the multi-purpose solutions [45,53].

In this study, six CL care solutions, mainly based on hydrogen peroxide, were retested according to the EN ISO 14729 [47]. There was special focus on creating a realistic setting by adding an organic load (artificial tear fluid) to the test conditions.

## Methods

### Contact lens care solutions

The CL care solutions listed in Table 1 were used according to the respective manufacturer’s instructions. The CL care solutions were assessed before their stated expiration dates and were taken from their original packaging.

**Table 1:** Contact lens care solutions

**trade name**	**manufacturer/ distributor**	**active ingredient**	**MMRDT**	**overnight**	**inactivation solution**
AOSEPT PLUS	Ciba Vision	3.0% H_2_O_2_	6 h	8 h	Eye See neutralising solution*
BlueVision	Ciba Vision	3.0% H_2_O_2_	6 h	8 h	Eye See neutralising solution*
Easy Sept	Bausch & Lomb	3.0% H_2_O_2_	6 h	8 h	Eye See neutralising solution*
Oxysept Comfort	AMO	3.0% H_2_O_2_	6 h	8 h	Eye See neutralising solution*
Optifree Replenish	Alcon	0.001% polyquad (polyquaternium-1), 0.0005% aldox (myristamidopropyl dimethylamine)	6 h	8 h	IA II**
Solocare Aqua	Ciba Vision	0.0001% polyhexanide	4 h	8 h	IA II**

MMRDT - manufacturer’s minimum recommended disinfection time.

* Eye See neutralising solution, Lapis Lazuli Int. NV: catalase, 0.01% EDTA, 0.002% merthiolate, isotonic buffer solution.

** inactivation solution II: 3% TSB (Roth, Karlsruhe, Germany), 3% polysorbate 80 (Serva, Heidelberg, Germany), 3% saponin (Fluka, Buchs, Switzerland), 0.1% L-histidine (Serva, Heidelberg, Germany), 0.1% L-cysteine (Merck, Darmstadt, Germany) in 1 L deionised water.

### Microorganisms

The test organisms *Pseudomonas aeruginosa* (ATCC 9027), *Staphylococcus aureus* (ATCC 6538), *Serratia marcescens* (ATCC 13880), *Candida albicans* (ATCC 10231) and *Fusarium solani* (ATCC 36031) were grown according to EN ISO 14729 (2001) [47].

### Artificial tear fluid

The artificial tear fluid was prepared by adding 0.5% of the tear-specific protein lysozyme (chicken egg lysozyme, Sigma Aldrich, Steinheim, Germany) and 0.1% of mucine (from porcine stomach, Sigma Adrich, Steinheim, Germany) to human blood serum. Human blood serum was used because of its similarity to natural tear fluid in terms of pH, osmolarity, ionic strength, and protein composition [54-57]. The serum was obtained from healthy blood donors at the Department of Transfusion Medicine of the University of Greifswald (Germany). Donors gave informed consent to provide an additional blood sample of 8 ml whole blood for research purposes. Fresh serum samples were collected daily in 10 ml tubes and used immediately. The amount of artificial tear fluid required for the quantitative suspension tests to simulate realistic conditions was determined by using a dry-weight method: hydrogel CLs were dried for 4 h using a desiccator and were weighed afterwards. The dried CLs were immersed in artificial tear fluid for 8 h, then dried and weighed again. The mean value of the calculated differences was approximately 0.1 g for two CLs. Therefore, 0.1 ml artificial fluid was determined for use in further quantitative suspension tests.

### Quantitative suspension test method

The quantitative suspension tests were performed in accordance with EN ISO 14729 (2001) [47]. 0.1 ml of broth culture (for bacteria ca. 10^9^ CFU/ml, for yeast ca. 10^8^ CFU/ml) was transferred into 10 ml CL care solution and incubated at 25°C for the manufacturer’s minimum recommended disinfection time (MMRDT) as well as overnight (8 h) (Table 1). In a parallel series, all experiments were performed likewise with the addition of 0.1 ml artificial tear fluid as the organic load.

After disinfection, the active ingredients were neutralised for 30 minutes at room temperature by transferring 1 ml of the incubated CL care solution into 9 ml of inactivation solution, which was individually assessed for each tested product (see Table 1). Afterwards, the serial dilutions were placed on the appropriate agar according to EN ISO 14729 [47]. Colonies were counted after 24 h (for bacteria and *C. albicans*) of incubation at 37°C and after 14 d (for *F. solani*) of incubation at 25°C. Colony counting allowed calculation of the original viable bacterial cell concentration in log [CFU/ml]); the results were reported as reduction factors of the log transformation data (RF log [CFU/ml]) by subtracting the CL care solution data from the control data. All experiments were performed for at least three different batches. According to EN ISO 14729 [47], the mean value of the reduction factors of these three batches was calculated. If the reduction factor of a batch was not an absolute value, e.g., ≥ 5.0 log [CFU/ml], this data was assumed to be the absolute value for the calculation, i.e., 5.0 log [CFU/ml]. If the results differed by more than 0.5 log [CFU/ml] from the mean value, the experiments were repeated. All in controls demanded by the norm were fulfilled.

### Statistical analyses

Mean values and standard deviations of reduction factors are reported as descriptive statistics. For each CL care solution and for each tested microorganism, the proportion of reduction factors exceeding pre-specified thresholds were calculated. The thresholds were determined according to the criteria specified in the EN ISO 14729 Stand Alone Test recommendations [47]: ≥ 3.0 log [CFU/ml] for bacteria and ≥ 1.0 log [CFU/ml] for fungi. The thresholds ≥ 5.0 log [CFU/ml] for bacteria according to the criteria in the norms EN 1040 [58], EN 13727 [59] and EN 1276 [60] and ≥ 4.0 log [CFU/ml] for fungi according to the criteria in the norms EN 1275 [61], EN 13624 [62] and EN 1650 [63] were additionally assessed for comparative purposes. The criteria of the different norms were considered as met if the thresholds were attained to at least 50%. Differences between proportions of reduction factors for CL care solutions were tested using the chi-square (*χ*^2^) test. In addition to analyses of the complete data set (1), subgroup analyses of tests with vs. without organic load (2) and MMRDT vs. overnight (3) were performed. P-values ≤ 0.05 were considered statistically significant.

## Results

**Figure 1:**
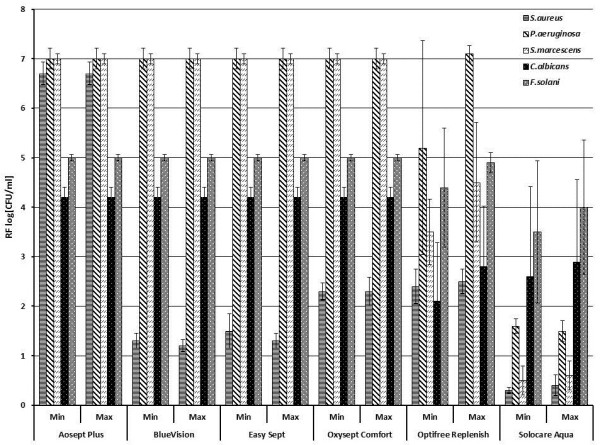
**Reduction factors of six different contact lens care solutions tested according to the Stand Alone Test (quantitative suspension test) of the EN ISO 14729 without organic load.** (Bars show the reduction factor of each contact lens care solution for each microorganism: *Staphylococcus aureus* (horizontally-striped bars), *Pseudomonas aeruginosa* (fasciated bars), *Serratia marcescens* (hatched bars), *Candida albicans* (dotted black bars) and *Fusarium solani* (dotted grey bars). Results are the mean values of the reduction factors in log [CFU/ml**]** ± standard deviation (error bars) of at least three different batches. Min = MMRDT (manufacturer’s minimum recommended disinfection time), Max = overnight (8h)).

**Figure 2:**
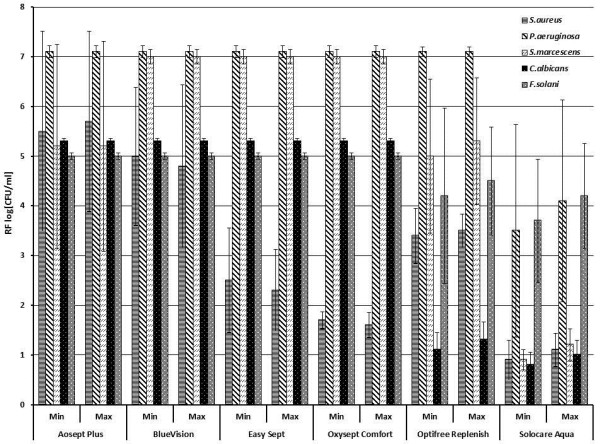
**Reduction factors of six different contact lens care solutions tested according to the Stand Alone Test (quantitative suspension test) of the EN ISO 14729 with organic load.** (Bars show the reduction factor of each contact lens care solution for each microorganism: *Staphylococcus aureus* (horizontally-striped bars), *Pseudomonas aeruginosa* (fasciated bars), *Serratia marcescens* (hatched bars), *Candida albicans* (dotted black bars) and *Fusarium solani* (dotted grey bars). (Results are the mean values of the reduction factors in log [CFU/ml**]** ± standard deviation of at least three different batches. Min = MMRDT (manufacturer’s minimum recommended disinfection time), Max = overnight (8h)).

The disinfection efficacies of the six different CL care solutions (Table 1) are reported in Table 2 without and in Table 3 with artificial tear fluid as the organic load, and additionally in Figure 1 without and in Figure 2 with organic load. All these data are the mean values of RF with standard deviation. Further, the calculated proportions of RFs exceeding the pre-specified thresholds are reported in Table 4 for each CL care solution and each microorganism. Table 5 shows these proportions without organic load and Table 6 with organic load. Reduction factor results by the different disinfecting times MMRDT and overnight are shown in Tables 7 and 8, respectively.

**Table 2:** Mean values of reduction factors of six different contact lens care solutions in log [CFU/ml] without organic load. Standard deviations are given in parentheses

**CL care solution**	**Time**	***S. aureus***	***P. aeruginosa***	***S. marcescens***	***C. albicans***	***F. solani***
**Aosept Plus**	6 h	> 6.7 (0.23)	> 7.0 (0.21)	> 7.0 (0.10)	> 4.2 (0.20)	> 5.0 (0.06)
	8 h	> 6.7 (0.23)	> 7.0 (0.21)	> 7.0 (0.10)	> 4.2 (0.20)	> 5.0 (0.06)
**BlueVision**	6 h	1.3 (0.15)	> 7.0 (0.21)	> 7.0 (0.10)	> 4.2 (0.20)	> 5.0 (0.06)
	8 h	1.2 (0.12)	> 7.0 (0.21)	> 7.0 (0.10)	> 4.2 (0.20)	> 5.0 (0.06)
**Easy Sept**	6 h	1.5 (0.35)	> 7.0 (0.21)	> 7.0 (0.10)	> 4.2 (0.20)	> 5.0 (0.06)
	8 h	1.3 (0.15)	> 7.0 (0.21)	> 7.0 (0.10)	> 4.2 (0.20)	> 5.0 (0.06)
**Oxysept Comfort**	6 h	2.3 (0.17)	> 7.0 (0.21)	> 7.0 (0.10)	> 4.2 (0.20)	> 5.0 (0.06)
	8 h	2.3 (0.29)	> 7.0 (0.21)	> 7.0 (0.10)	> 4.2 (0.20)	> 5.0 (0.06)
**Optifree Replenish**	6 h	2.4 (0.35)	5.2 (2.16)	3.5 (0.66)	2.1 (1.18)	4.4 (1.20)
	8 h	2.5 (0.25)	> 7.1 (0.17)	4.5 (1.21)	2.8 (1.22)	> 4.9 (0.20)
**Solocare Aqua**	4 h	0.3 (0.06)	1.6 (0.15)	0.5 (0.29)	2.6 (1.82)	3.5 (1.44)
	8 h	0.4 (0.21)	1.5 (0.21)	0.6 (0.29)	2.9 (1.65)	4.0 (1.35)

**Table 3:** Mean values of reduction factors of six different contact lens care solutions in log [CFU/ml] with organic load. Standard deviations are given in parentheses

**CL care solution**	**Time**	***S. aureus***	***P. aeruginosa***	***S. marcescens***	***C. albicans***	***F. solani***
**Aosept Plus**	6 h	5.5 (2.01)	> 7.1 (0.12)	5.2 (2.05)	> 5.3 (0.06)	> 5.0 (0.06)
	8 h	5.7 (1.81)	> 7.1 (0.12)	5.2 (2.11)	> 5.3 (0.06)	> 5.0 (0.06)
**BlueVision**	6 h	5.0 (1.39)	> 7.1 (0.12)	> 7.0 (0.15)	> 5.3 (0.06)	> 5.0 (0.06)
	8 h	4.8 (1.64)	> 7.1 (0.12)	> 7.0 (0.15)	> 5.3 (0.06)	> 5.0 (0.06)
**Easy Sept**	6 h	2.5 (1.05)	> 7.1 (0.12)	> 7.0 (0.15)	> 5.3 (0.06)	> 5.0 (0.06)
	8 h	2.3 (0.82)	> 7.1 (0.12)	> 7.0 (0.15)	> 5.3 (0.06)	> 5.0 (0.06)
**Oxysept Comfort**	6 h	1.7 (0.17)	> 7.1 (0.12)	> 7.0 (0.15)	> 5.3 (0.06)	> 5.0 (0.06)
	8 h	1.6 (0.25)	> 7.1 (0.12)	> 7.0 (0.15)	> 5.3 (0.06)	> 5.0 (0.06)
**Optifree Replenish**	6 h	3.4 (0.55)	> 7.1 (0.10)	5.0 (1.55)	1.1 (0.35)	4.2 (1.76)
	8 h	3.5 (0.34)	> 7.1 (0.10)	5.3 (1.28)	1.3 (0.36)	4.5 (1.08)
**Solocare Aqua**	4 h	0.9 (0.40)	3.5 (2.24)	0.9 (0.20)	0.8 (0.25)	3.7 (1.24)
	8 h	1.1 (0.34)	4.1 (2.03)	1.2 (0.32)	1.0 (0.30)	4.2 (1.06)

**Table 4:** Proportions of reduction factors exceeding pre-specified thresholds for each CL care solution and for each tested microorganism / complete data set

	***S. aureus***				***P. aeruginosa***				***S. marcescens***				***C. albicans***				***F. solani***			
	**RF ≥ 3**		**RF ≥ 5**		**RF ≥ 3**		**RF ≥ 5**		**RF ≥ 3**		**RF ≥ 5**		**RF ≥ 1**		**RF ≥ 4**		**RF ≥ 1**		**RF ≥ 4**	
	**%**	**N**	**%**	**N**	**%**	**N**	**%**	**N**	**%**	**N**	**%**	**N**	**%**	**N**	**%**	**N**	**%**	**N**	**%**	**N**
**AOSEPT PLUS**	88.9	18	77.8	18	100	12	100	12	88.9	18	66.7	18	100	12	100	12	100	12	100	12
**BlueVision**	61.1	18	22.2	18	100	12	100	12	100	12	100	12	100	12	100	12	100	12	100	12
**Easy Sept**	11.1	18	0	18	100	12	100	12	100	12	100	12	100	12	100	12	100	12	100	12
**Oxysept Comfort**	0	12	0	12	100	12	100	12	100	12	100	12	100	12	100	12	100	12	100	12
**Optifree Replenish**	61.1	18	0	18	94.4	18	83.3	18	95.8	24	20.8	24	94.4	18	16.7	18	95.8	24	87.5	24
**Solocare Aqua**	0	18	0	18	38.9	18	11.1	18	0	12	0	12	77.8	18	22.2	18	100	24	58.3	24
***χ***^**2**^**test**	< 0.001		< 0.001		< 0.001		< 0.001		< 0.001		< 0.001		0.041		< 0.001		0.695		< 0.001	

The intervals ≥ 3.0 log [CFU/ml] for bacteria and ≥ 1.0 log [CFU/ml] for fungi were assessed according to the criteria of the EN ISO 14729 Stand Alone Test [47]. The intervals ≥ 5.0 log [CFU/ml] for bacteria according to the criteria of the norms EN 1040 [58], EN 13727 [59] and EN 1276 [60] and ≥ 4.0 log [CFU/ml] for fungi according to the criteria of the norms EN 1275 [61], EN 13624 [62] and EN 1650 [63] were assessed.

**Table 5:** Proportions of reduction factors exceeding pre-specified thresholds for each CL care solution and for each tested microorganism / without organic load

	***S. aureus***				***P. aeruginosa***				***S. marcescens***				***C. albicans***				***F. solani***			
	**RF ≥ 3**		**RF ≥ 5**		**RF ≥ 3**		**RF ≥ 5**		**RF ≥ 3**		**RF ≥ 5**		**RF ≥ 1**		**RF ≥ 4**		**RF ≥ 1**		**RF ≥ 4**	
	**%**	**N**	**%**	**N**	**%**	**N**	**%**	**N**	**%**	**N**	**%**	**N**	**%**	**N**	**%**	**N**	**%**	**N**	**%**	**N**
**AOSEPT PLUS**	100	6	100	6	100	6	100	6	100	6	100	6	100	6	100	6	100	6	100	6
**BlueVision**	0	6	0	6	100	6	100	6	100	6	100	6	100	6	100	6	100	6	100	6
**Easy Sept**	0	6	0	6	100	6	100	6	100	6	100	6	100	6	100	6	100	6	100	6
**Oxysept Comfort**	0	6	0	6	100	6	100	6	100	6	100	6	100	6	100	6	100	6	100	6
**Optifree Replenish**	0	6	0	6	91.7	12	75	12	91.7	12	8.3	12	100	12	25	12	100	12	91.7	12
**Solocare Aqua**	0	6	0	6	0	6	0	6	0	6	0	6	91.7	12	33.3	12	100	12	58.3	12
***χ***^**2**^**test**	< 0.001		< 0.001		< 0.001		< 0.001		< 0.001		< 0.001		0.690		< 0.001		1		0.024	

The intervals ≥ 3.0 log [CFU/ml] for bacteria and ≥ 1.0 log [CFU/ml] for fungi were assessed according to the criteria of the EN ISO 14729 Stand Alone Test [47]. The intervals ≥ 5.0 log [CFU/ml] for bacteria according to the criteria of the norms EN 1040 [58], EN 13727 [59] and EN 1276 [60] and ≥ 4.0 log [CFU/ml] for fungi according to the criteria of the norms EN 1275 [61], EN 13624 [62] and EN 1650 [63] were assessed.

**Table 6:** Proportions of reduction factors exceeding pre-specified thresholds for each CL care solution and for each tested microorganism / with organic load

	***S. aureus***				***P. aeruginosa***				***S. marcescens***				***C. albicans***				***F. solani***			
	**RF ≥ 3**		**RF ≥ 5**		**RF ≥ 3**		**RF ≥ 5**		**RF ≥ 3**		**RF ≥ 5**		**RF ≥ 1**		**RF ≥ 4**		**RF ≥ 1**		**RF ≥ 4**	
	**%**	**N**	**%**	**N**	**%**	**N**	**%**	**N**	**%**	**N**	**%**	**N**	**%**	**N**	**%**	**N**	**%**	**N**	**%**	**N**
**AOSEPT PLUS**	83.3	12	66.7	12	100	6	100	6	83.3	12	50	12	100	6	100	6	100	6	100	6
**BlueVision**	91.7	12	33.3	12	100	6	100	6	100	6	100	6	100	6	100	6	100	6	100	6
**Easy Sept**	16.7	12	0	12	100	6	100	6	100	6	100	6	100	6	100	6	100	6	100	6
**Oxysept Comfort**	0	6	0	6	100	6	100	6	100	6	100	6	100	6	100	6	100	6	100	6
**Optifree Replenish**	91.7	12	0	12	100	6	100	6	100	12	33.3	12	83.3	6	0	6	91.7	12	83.3	12
**Solocare Aqua**	0	12	0	12	58.3	12	16.7	12	0	6	0	6	50	6	0	6	100	12	58.3	12
***χ***^**2**^**test**	< 0.001		< 0.001		0.014		<0.001		< 0.001		< 0.001		0.03		< 0.001		0.69		0.047	

The intervals ≥ 3.0 log [CFU/ml] for bacteria and ≥ 1.0 log [CFU/ml] for fungi were assessed according to the criteria of the EN ISO 14729 Stand Alone Test [47]. The intervals ≥ 5.0 log [CFU/ml] for bacteria according to the criteria of the norms EN 1040 [58], EN 13727 [59] and EN 1276 [60] and ≥ 4.0 log [CFU/ml] for fungi according to the criteria of the norms EN 1275 [61], EN 13624 [62] and EN 1650 [63] were assessed.

**Table 7:** Proportions of reduction factors exceeding pre-specified thresholds for each CL care solution and for each tested microorganism after MMRDT

	***S. aureus***				***P. aeruginosa***				***S. marcescens***				***C. albicans***				***F. solani***			
	**RF ≥ 3**		**RF ≥ 5**		**RF ≥ 3**		**RF ≥ 5**		**RF ≥ 3**		**RF ≥ 5**		**RF ≥ 1**		**RF ≥ 4**		**RF ≥ 1**		**RF ≥ 4**	
	**%**	**N**	**%**	**N**	**%**	**N**	**%**	**N**	**%**	**N**	**%**	**N**	**%**	**N**	**%**	**N**	**%**	**N**	**%**	**N**
**AOSEPT PLUS**	88.9	9	77.8	9	100	6	100	6	88.9	9	66.7	9	100	6	100	6	100	6	100	6
**BlueVision**	66.7	9	22.2	9	100	6	100	6	100	6	100	6	100	6	100	6	100	6	100	6
**Easy Sept**	11.1	9	0	9	100	6	100	6	100	6	100	6	100	6	100	6	100	6	100	6
**Oxysept Comfort**	0	6	0	6	100	6	100	6	100	6	100	6	100	6	100	6	100	6	100	6
**Optifree Replenish**	55.6	9	0	9	88.9	9	66.7	9	91.7	12	16.7	12	88.9	9	11.1	9	91.7	12	83.3	12
**Solocare Aqua**	0	9	0	9	33.3	9	11.1	9	0	6	0	6	66.7	9	22.2	9	100	12	50	12
***χ***^**2**^**test**	< 0.001		< 0.001		0.001		< 0.001		< 0.001		< 0.001		0.132		< 0.001		0.69		0.013	

The intervals ≥ 3.0 log [CFU/ml] for bacteria and ≥ 1.0 log [CFU/ml] for fungi were assessed according to the criteria of the EN ISO 14729 Stand Alone Test [47]. The intervals ≥ 5.0 log [CFU/ml] for bacteria according to the criteria of the norms EN 1040 [58], EN 13727 [59] and EN 1276 [60] and ≥ 4.0 log [CFU/ml] for fungi according to the criteria of the norms EN 1275 [61], EN 13624 [62] and EN 1650 [63] were assessed.

**Table 8:** Proportions of reduction factors exceeding pre-specified thresholds for each CL care solution and for each tested microorganism after overnight disinfection

	***S. aureus***				***P. aeruginosa***				***S. marcescens***				***C. albicans***				***F. solani***			
	**RF ≥ 3**		**RF ≥ 5**		**RF ≥ 3**		**RF ≥ 5**		**RF ≥ 3**		**RF ≥ 5**		**RF ≥ 1**		**RF ≥ 4**		**RF ≥ 1**		**RF ≥ 4**	
	**%**	**N**	**%**	**N**	**%**	**N**	**%**	**N**	**%**	**N**	**%**	**N**	**%**	**N**	**%**	**N**	**%**	**N**	**%**	**N**
**AOSEPT PLUS**	88.9	9	77.8	9	100	6	100	6	88.9	9	66.7	9	100	6	100	6	100	6	100	6
**BlueVision**	55.6	9	22.2	9	100	6	100	6	100	6	100	6	100	6	100	6	100	6	100	6
**Easy Sept**	11.1	9	0	9	100	6	100	6	100	6	100	6	100	6	100	6	100	6	100	6
**Oxysept Comfort**	0	6	0	6	100	6	100	6	100	6	100	6	100	6	100	6	100	6	100	6
**Optifree Replenish**	66.7	9	0	9	100	9	100	9	100	12	25	12	100	9	22.2	9	100	12	91.7	12
**Solocare Aqua**	0	9	0	9	44.4	9	11.1	9	0	6	0	6	88.9	9	22.2	9	100	12	66.7	12
***χ***^**2**^**test**	< 0.001		< 0.001		0.001		<0.001		< 0.001		< 0.001		0.585		< 0.001		1		0.087	

The intervals ≥ 3.0 log [CFU/ml] for bacteria and ≥ 1.0 log [CFU/ml] for fungi were assessed according to the criteria of the EN ISO 14729 Stand Alone Test [47]. The intervals ≥ 5.0 log [CFU/ml] for bacteria according to the criteria of the norms EN 1040 [58], EN 13727 [59] and EN 1276 [60] and ≥ 4.0 log [CFU/ml] for fungi according to the criteria of the norms EN 1275 [61], EN 13624 [62] and EN 1650 [63] were assessed.

The primary criteria of the Stand Alone Test recommended in EN ISO 14729 [47] require a RF of ≥ 3 log units for bacteria and ≥ 1 log unit for fungi. The results show that only Aosept Plus met these criteria and even exceeded them with > 6 log units for bacteria and > 4 log units for fungi without organic load (Tables 2 and 5). With organic load, the product yields lower yet sufficient RFs of > 5 for *S. aureus* and *S. marcescens*, and unchanged high RFs for all other microorganisms. The harmonised requirements for determining the efficacies of chemical disinfectants and antiseptics, i.e., a RF of ≥ 5 log units for bacteria and ≥ 4 log units for fungi, are solely met by Aosept Plus regardless of the test conditions (Tables 4, 5, 6, 7, 8).

Although Blue vision, Easy Sept and Oxysept Comfort also contain 3% hydrogen peroxide, these three products yielded different results. BlueVision does not pass the Stand Alone criteria due to insufficient efficacy against *S. aureus*. The efficacy against the other microorganisms is particularly high, with a RF > 7 log units for bacteria and > 4 log unit for fungi. In the presence of organic load, the efficacy of BlueVison against *S. aureus* increases dramatically, while that against all other microoraganisms is not affected by the organic load in any way. The great increase of efficacy against *S. aureus* enables the product to pass the Stand Alone criteria in the presence of organic load. The criteria of the harmonised norms for chemical disinfectants and antiseptics, however, are still not met.

Without organic load, Easy Sept and Oxysept Comfort exhibited behaviour similar to BlueVision, showing a lack of efficacy against *S. aureus* and therefore not passing the Stand Alone Test either. The positive effect of organic load on the efficacy of BlueVision against *S. aureus* can only be be slightly observed for Easy Sept. The efficacy of Oxysept Comfort decreases even further. This means that Easy Sept and Oxysept Comfort do not fulfill the requirements of the Stand Alone Test, either with or without organic load. Consequently, the criteria of the harmonised norms for chemical disinfectants and antiseptics also cannot be met.

Although the RFs for the polyquad-based CL care product Optifree Replenish are generally lower than those of the hydrogen-peroxide-based products, the requirements of > 3 log units for bacteria and > 1 log unit for fungi are met in the presence of organic load. This again shows how the organic load can have a positive influence on the efficacy of the product. Without organic load, the product exhibits mostly lower RFs for bacteria than with organic load and in the case of *S. aureus* fails to meet the test criteria.

Solocare Aqua, the polyhexanide-based product, exhibits the lowest RFs in all cases. The product passes the Stand Alone Test neither with nor without organic load. Nevertheless, the influence of organic load, can be observed for Solocare Aqua as well: especially the efficacy against *P. aeruginosa* increases greatly by more than 2 log units. On *C. albicans,* the organic load has a noticeable negative influence, reducing the efficacy by nearly 2 log units.

Neither of the multi-purpose solutions (MPSs), Optifree Replenish and Solocare Aqua, meet the criteria of the harmonised norms for chemical disinfectants and antiseptics.

The prolonged disinfection time (overnight) did not noticeably influence the disinfection efficacy compared to the MMRDT for any of the tested CL care solutions. The *χ*^2^ test shows that in most cases, the disinfection efficacies significantly differ between the different CL care solutions.

## Discussion

Many studies in the past have determined the disinfecting efficacy of polyhexanide- or polyquad-based multi-purpose solutions (MPSs) and hydrogen-peroxide-based CL care solutions according to the primary criteria of the Stand Alone Test of the EN ISO 14729 [41,45,50-53,64-73]. Kramer et al. [45] and other authors [53,65,73] investigated the disinfecting efficacy of various CL care solutions including MPSs and hydrogen-peroxide-based CL care solutions, and reported that the hydrogen-peroxide-based CL care solution Aosept Plus proved to be the most effective product, yielding reduction factors of > 5 log units. This study also confirms that Aosept Plus, a 3% hydrogen-peroxide-based CL care solution, yields the best results independent of all influencing factors, meets the primary criteria of the Stand Alone Test, and furthermore fulfills the harmonised requirements for determining the efficacies of chemical disinfectants and antiseptics. For more than 20 years now, it is known that CL care solutions based on 3% hydrogen peroxide are mostly effective against a broad range of microorganisms [74,75] also in practical settings [76,77], but it is also known that their efficacies can vary [45,53]. This study found differences in the efficacies against *S. aureus*, although the four tested hydrogen peroxide solutions have identical concentrations of active ingredient. The same effect has been observed for MPSs which are based on the same amount of active ingredient, for example 0.0001% polyhexanide [45,53,73]. Therefore, further ingredients such as salts, buffers, surfactants, sequestering agents, or wetting agents as well as the physical and chemical characteristics of the tested CL care solutions must be major influencing factors and must have the ability to increase the disinfection efficacies of CL care solutions [12,69,78-81]. Thus, the active ingredient itself is not a guarantee for sufficient disinfecting performance, and proof of efficacy by meeting a harmonised norm is essential.

Comparing MPSs, it has often been reported that polyquad-based CL care solutions such as Optifree are more effective than polyhexanide-based CL care solutions [41,53,70,72], which is in line with the findings of this study. In particular Solocare Aqua seems to possess hardly any antimicrobial and antifungal characteristics [53]. These findings are in agreement with those of a recent *in-vivo* study by Nzeako et al. [12], who examined the microbial contamination of various commonly used CL care solutions. MPSs, especially those based on polyhexanide or related active ingredients, were found to be contaminated with Gram-negative bacteria, in particular *P. aeruginosa*, yeasts (Candida spp.), moulds, and Gram-positive bacteria (especially *S. marcescens*) as well. The high number of contaminated MPSs reported in *in-vivo* studies [12,25,45,46] and the association of MPSs with an increased risk of CL-related microbial keratitis [38-44] suggest that MPSs – especially those based on polyhexanide or related active ingredients –should be improved to yield higher antimicrobial and antifungal characteristics, and therefore should be tested under more realistic conditions before being placed on the market.

Especially the influence of the tear fluid and its components is an important factor for simulating more realistic conditions. Thus, different studies have reappraised the effect of organic load on test results [45,53,72,82], for example, 0.2% bovine albumin [45], 0.2% mucine [73] or even more contaminated conditions with 1% albumin and 0.1% mucine [53]. All these studies exhibit similar results; organic load has a negative effect on the disinfecting efficacy of a number of CL care solutions. Still another organic load was used in this study: a special artificial tear fluid consisting of human blood serum, lysozyme and mucine to mimic realistic test settings. Although human blood serum represents a useful analogue to human tear fluid, serum has a higher protein concentration, lower quantities of antimicrobial substances, and lacks tear-specific proteins. Therefore, the tear-specific proteins lysozyme and mucine were added. This study revealed that in the presence of the described artificial tear fluid, the disinfecting efficacy against *S. aureus* surprisingly increases for most CL care solutions, and in particular for BlueVision and Optifree Replenish, which then pass the primary criteria of the Stand Alone Test. This increased efficacy has also been observed for Solocare Aqua against *P. aeruginosa*, whereas the presence of organic load seems to have no effect on the efficacies against *S. marcescens* and *F. solani*. In contrast, the yeast *C. albicans* was more resistant to the MPSs in the presence of the artificial tear fluid. This unequal effect on the disinfecting efficacies of the different CL care solutions can possibly be explained by the antimicrobial characteristics of lysozyme and other proteins in the artificial tear fluid. Lysozyme, also called *N*-acetylmuramidase, is an enzyme with antimicrobial activity against a wide range of microorganisms [83-88]. This enzyme catalyses the hydrolysis of 1.4-*β*-bonds between *N*-acetylmuramic acid (NAM) and *N*-acetylglucosamine (NAG) residues in the peptidoglycan polymers of bacterial cells, also called murein, resulting in the lysis of the sensitive bacterial cells [83]. Mostly Gram-positive bacteria, such as *S. aureus*, are susceptible to lysozyme due to their freely accessible murein cell wall [83]. It has been reported that the antibacterial effect of lysozyme can vary between different bacterial species and strains and that it can be influenced by additional substances (lactoferrin, EDTA etc.) or changes in environmental conditions (pH value, temperature, pressure, etc.) [83-88]. These influences may explain the findings in this study: the noticeably increased efficacy against the Gram-positive bacterium *S. aureus*, the slightly increased or lack of efficacy against the Gram-negative bacteria *P. aeruginosa* and *S. marcescens,* respectively. Through the observation that the artificial tear fluid used in this study influences the disinfecting efficacy of CL care solutions, especially that of MPSs, in a different way than do albumin, mucine or even the organic load suggested in EN ISO 14729, it becomes obvious that the test conditions in the EN ISO 14729 should be revised to create more realistic conditions, e.g., by using a more realistic artificial tear fluid.

Since the EN ISO 14729 was adopted in 2001, Kramer et al. [45] have criticised the following points: i. efficacy testing with organic load is only mentioned as an option and the type of organic load is not specified; ii. for bacteria, the test protocol neither includes data on the standard deviation nor on the number of test reproductions necessary for statistical significance; iii. a RF of 1 log unit for *C. albicans* and *F. solani* cannot be exactly ascertained experimentally and falls within the scatter range of the quantitative suspension test (± 0.5 log); iv. neutralisation testing is not adequately defined and must be described more precisely; v. testing against *Acanthamoeba* species is not recommended but should be defined with a RF of at least > 2 log units; vi. compared to other harmonised norms, the required RFs are too for evaluating the microbicidal efficacy of disinfectants; vii. a test under practical conditions (Regimen Test) is only required if the primary criteria of the Stand Alone Test are not met. Further critical points must be considered when testing CL care solution: i. laboratory strains may be inadequate for assuring that the CL care solutions will be equally effective against clinical isolates [41,66,68,89-91].; ii. After extended periods of storage, the disinfecting efficacy of polyhexanide-based MPSs may decrease due to the accumulation of polyhexanide on the CL material or CL case [70,92]; iii. higher storage temperatures can lead to decreased disinfecting efficacies [93]; longer storage times of open hydrogen-peroxide-based CL care solutions can lead to a decrease of the hydrogen peroxide concentration and thus decreased disinfecting efficacy.

Bearing a number of these influences in mind, some changes were incorporated into the EN ISO 14729 [94] in 2010: i. clarification of the criteria of the Stand Alone Test for moulds (CL care solution direct test); ii. changes in the recommendations regarding the rub-and-/or-rinse steps to the effect that if the CL care solution can be used without a rub and-/or-rinse step according to the manufacturer’s instructions, the CL care solution must pass the secondary criteria of the Stand Alone Test as well as the criteria of the Regimen Test in the presence of organic load; iii. incorporation of requirements for testing CL care solutions for use on silicone hydrogel CLs. Despite these changes, the aspects which have been criticised in the past still have not been adequately addressed in this amendment. Especially the demands for i. stricter reduction factors, ii. a quantitative suspension with and without organic load in combination with a test simulating practical conditions, and iii. a specified and realistic artificial tear fluid as organic load have been neglected.

In the European test hierarchy for chemical disinfectants and antiseptics, stricter reduction factors of ≥ 5 log units for bacteria and ≥ 4 log units for fungi are recommended in each test norm regardless of the type of test (quantitative suspension test or practical setting). These harmonised requirements could also be adequate criteria for the determination of the efficacies of CL care solutions. Furthermore, the European test hierarchy is a system of three tests: phase 1 (quantitative suspension test without organic load); phase 2 step 1 (quantitative suspension test with organic load); phase 2 step 2 (carrier test - practical settings). All three tests must be performed and passed in order to determine the bactericidal and fungicidal efficacies of chemical disinfectants and antiseptics. This system is another improvement that could be made in the testing of CL care solutions. Moreover, improvements to the Regimen Test as a phase 2 steps 2 test have recently been suggested in order to provide a more realistic evaluation of applicable CL care disinfecting solutions [95].

## Conclusions

Because artificial tear fluid influences the disinfecting efficacy of contact lens care solutions, especially that of multi-purpose solutions, in a different way than does albumin, mucine, or the organic load suggested in EN ISO 14729, it becomes obvious that the test conditions in the EN ISO 14729 should be revised in order to create more realistic conditions, e.g., by using a more realistic artificial tear fluid. Furthermore, we suggest adapting the EN ISO 14729 to the European test hierarchy for chemical disinfectants and antiseptics, which consists of three test phases and also requests meeting stricter criteria in order to pass the test.

The CL industry itself has also started to respond to the criticism by enhancing their CL care products, e.g., AMO Eyecare with its product COMPLETE® RevitaLens, Alcon’s Opti-Free EverMoist or Bausch & Lomb’s Biotrue^TM^. These three products have an increased concentration of active ingredients, combinations of active ingredients have been introduced (polyquad together with another active ingredient such as a biguanide or an amidoamine), and the recommendation to perform an adequate rub-and-rinse step has been revived. However, it is not expected that the compliance rate of CL wearers with their CL care regimen will increase considerably, and, therefore, the criteria to evaluate the disinfecting efficacy of CL care products must be made substantially more rigorous and a certain safety margin should be included. Critical CL care products would then disappear from the market leaving those products that exhibit an adequate disinfecting efficacy. This way it is possible to counteract the careless behaviour of the great majority of CL wearers and the risk of CL-related microbial keratitis and other infections will be decreased.

## Competing interests

The authors declare that they have no competing interests.

## Authors' contributions

CH and AK designed the study. AK coordinated the study. DW performed the quantitative suspension tests. TK was responsible for statistical analysis and interpretation of the data. CH wrote the manuscript and CH, DW, TK and AK were involved in drafting the manuscript and revising it critically for important intellectual content. All authors have read and approved the final manuscript.

## Pre-publication history

The pre-publication history for this paper can be accessed here:

http://www.biomedcentral.com/1471-2334/12/241/prepub
